# In-store beverage pricing and marketing before and after a sugar-sweetened beverage tax in Newfoundland and Labrador

**DOI:** 10.1017/S1368980026102146

**Published:** 2026-02-16

**Authors:** Kierra Dooley, Daniel A. Zaltz, Scott V. Harding, Kayla Crichton, David Hammond, Yanqing Yi, Marie-Claude Paquette, Peizhong Peter Wang, Kim D. Raine, Rachel Prowse

**Affiliations:** 1 Department of Human Biosciences, Memorial University of Newfoundland Faculty of Science, Canada; 2 School of Epidemiology and Public Health, Faculty of Medicine, University of Ottawa, Ottawa, Canada; 3 School of Public Health Sciences, University of Waterloo, Canada; 4 Division of Population Health and Applied Health Sciences, Memorial University of Newfoundland Faculty of Medicine, Canada; 5 Institut national de sante publique du Quebec, Canada; 6 Centre for New Immigrant Well-Being, Canada; 7 Dalla Lana School of Public Health, University of Toronto, Canada; 8 School of Public Health, University of Alberta, Canada

**Keywords:** Sugar-sweetened beverages, observational study, price, revenue, taxes, health promotion, marketing

## Abstract

**Objective:**

Newfoundland and Labrador (NL) introduced Canada’s first excise tax on sugar-sweetened beverages (SSB) in 2022. Industry marketing practices in response to SSB taxation may affect public health impacts. We examined changes in posted beverage pricing and marketing of taxable and non-taxable beverages in NL before and after the SSB tax was implemented.

**Design:**

Pre-/post-observational study with in-store audits of beverage prices and marketing. Changes including pricing discounts and promotions were assessed at the individual beverage level for pre/post-tax implementation years.

**Setting:**

Eighty food stores (grocery, convenience, drug and dollar) in NL, Canada.

**Results:**

There was no evidence of a change in posted shelf prices between pre/post years. There was a significant increase (+2·5 %, *χ* ^2^ = 9·693, *P* = 0·002) in proportion of discounted taxable SSB with no change in non-taxable beverages (*P* = 0·350). There were no significant differences in change of number of promotions for taxable SSB (+5·2 [−0·1, 10·5], *F* = 3·789, *P* = 0·053) nor non-taxable beverages (+3·4 [–1·0, 7·7], *F* = 2·268, *P* = 0·134).

**Conclusions:**

The lack of change in posted prices of taxable SSB indicates that the NL SSB tax was not communicated at the point of decision-making. While some marketing changes post-tax were observed, results should be interpreted cautiously as they cannot be attributed definitively to the tax. Existing literature implies that industry may adapt marketing conduct to counteract beverage taxes. Such changes were limited in NL, suggesting retailers may have opted not to display the tax rather than attempt to actively counteract it. Lack of transparency surrounding the tax may neutralise intended behavioural effects.

Sugar-sweetened beverages (SSB) are a substantial contributor to dietary added sugar intake globally.^(1)^ Reports from the 2015 Canadian Community Health Survey Nutrition Module confirm that sugary beverages (SSB and free sugar-containing beverages) represent the top source of sugars for all ages in the Canadian population, while regular soft drinks alone represent a top sugar source among older children and adults.^(2)^ Data from the 2015 Canadian Community Health Survey Nutrition Module show that Canadians consume an average of 204 mL of SSB per day, representing 36 % of beverage calories and contributing 99 kcal to their daily energy intake – more than any other type of beverage.^(3)^ Over forty different adverse health outcomes have been found to be significantly associated with dietary sugar consumption.^(1)^ Regular SSB consumption is linked to an increased risk of chronic disease,^(4)^ weight gain and obesity,^(5,6)^ metabolic syndrome,^(7)^ as well as dental issues.^(8)^

Taxation of SSB is strongly recommended by the WHO as a public health intervention to reduce SSB intake and in turn decrease sugar consumption and disease prevalence.^(9)^ One Canadian study estimates that a tax of $0·015/oz would reduce SSB consumption by 17 %.^(10)^ Over 100 countries have implemented SSB taxes, which have been associated with decreases in purchasing and consumption.^(11)^ Meta-analyses have found a 15 % mean reduction in SSB sales in response to taxes, with demand being highly sensitive to price increase.^(12)^

On 1 September 2022, Newfoundland and Labrador (NL) became the first Canadian province to introduce an excise tax on SSB – at a rate of $0·20 per liter to be imposed on wholesalers with the expectation of being passed down to the consumer – aiming to reduce SSB consumption and promote healthier beverage alternatives.^(13)^ The tax targets certain beverages with added sugars, such as soft drinks, fruit-flavored beverages and energy drinks, while excluding beverages such as 100 % fruit juices and those prepared at point of purchase.^(13)^ The tax was promoted as a health initiative to combat health concerns, relating to NL’s high chronic disease burden^(14)^ while also having the lowest life expectancy of all provinces.^(15)^ The tax was first announced in May 2021, with little guidance prior to tax introduction provided on implementation and the absence of any form of transition phases for industry.^(16)^

Responses to SSB taxes from industry, including changes in pricing, formulation and promotion, serve to complicate and mediate the influence of tax-induced price changes on consumption.^(17)^ The ‘four Ps’ of marketing – product, price, promotion and place – are used as a framework to describe industry responses though literature describing these changes post-tax implementation is scarce.^(18)^ SSB are among the most marketed product globally.^(19)^ Advertising across diverse platforms has been shown to drive consumption especially in young people, who are leading SSB consumers.^(20)^ Existent literature links soft drink marketing to changes in beverage preferences, choices and consumption.^(18,21,22)^ Studies have shown that strategic industry decisions surrounding SSB price (including tax pass-through), distribution strategies and communication are used in an attempt to mitigate impacts of SSB taxes on consumers.^(17)^ In-store marketing strategies include place and proximity strategies (e.g. increasing product placement at high-visibility and impulse purchasing areas), as well as promotions such as signage or discounted pricing.^(23)^ However, promotional changes are among the least studied aspects of industry response to SSB taxation.^(17)^ The majority of research on sugar taxes is based on modelling studies, though as they increase in popularity, observational studies are becoming increasingly possible.^(17)^

This study aimed to evaluate changes in beverage pricing and marketing (discount pricing, product placement and promotional signage) of taxable and non-taxable beverages in NL, Canada, before and after the implementation of its SSB tax on 1 September 2022.

## Methods

### Study design

This study is part of a multicomponent evaluation of the real-life impacts of the NL SSB tax. A series of outcomes were analysed to illustrate the impacts of the tax design and implementation; specifically, changes in beverage prices, promotion, purchasing and consumption. This study reports on food stores audits completed by trained research assistants documenting the beverage pricing, product placement and promotional signage in-store. The outcomes of interest are based on established principles of marketing strategies: product, price, promotion and place.^(24)^ This study is not intended as an evaluation of the total economic pass-through of the tax; rather it intends to evaluate the communication of the cost of the tax to consumers in the form of higher prices on posted shelf tags in-store. Unpublished research from our team confirms that a large majority of retailers are applying the SSB tax at the register during checkout, where it may appear in the tax line alongside other fees and taxes (e.g. carbon, environmental, electronic or bottle fees), rather than affecting the visible item cost directly. Lack of communication of the tax reflected in posted price at the point of decision-making reduces the likelihood that the tax will be effective as a financial disincentive for purchasing targeted beverages. Our investigation revealing that the SSB tax was not clearly displayed on shelf prices prompted further investigation into the pass-through of the SSB tax at check-out and display of the SSB tax on store receipts. A complete study on the implementation of the SSB tax is forthcoming, which will help to contextualise this research on price changes in-stores.

### Store sampling

The store sampling frame was obtained from a list of public food retailers provided by the Government of NL. Food retailers were coded using the North American Industry Classification System codes as grocery, convenience, drug and dollar stores. From a list of 202 stores (*n* 35 grocery, *n* 128 convenience, *n* 26 drug, and *n* 13 dollar), a census of all food retailers in St. John’s Census Metropolitan Area, we randomly selected a proportionate sample of 108 food stores, stratified by type and ownership (see Supplement A, Table A). Within the grocery and convenience store types, separately, we randomly selected half of the stores by ownership model, split by store name. For drug stores, we randomly selected one-third of stores by ownership model, split by store name. For dollar stores, we included all locations due to the smaller number of stores. A total of twenty grocery stores, sixty-two convenience stores, thirteen drug stores and thirteen dollar stores were sampled in the St. John’s metropolitan area. Another twenty-six rural stores (*n* 7 grocery stores and 19 convenience stores) from the Burin, Bonavista and Northern Peninsulas were randomly selected. A total of eighty retailers (*n* 18 grocery, *n* 50 convenience, *n* 7 drug stores, *n* 5 dollar stores and *n* 15 rural stores) were included in the final sample after stores with zero complete audits were removed due to store closures, store access denial and inadequate staff capacity to complete the audit during the data collection period (see Supplement A, Figure A). Our sample includes approximately half of all grocery stores and convenience stores and more than one-third of all drug stores and dollar stores in St. John’s and is approximately representative of the stores available in St. John’s. The rural stores are located more than 300 km from St. John’s and are in less accessible, more remote areas of the province.

### Data collection

In-store audits were conducted to gather data on posted beverage prices and marketing (price discounts, product placements and promotional signs). A total of nine rounds of in-store beverage data collection were completed by research assistants; five rounds prior to tax implementation (December 2021–August 2022) and four post-tax (October 2022–August 2023).

Using an NL-adapted Beverage Tax Food Store Observation Form,^(25)^ research assistants recorded posted shelf prices for up to fifty-three beverage products, depending on availability by store, across eight categories: soft drinks, sports drinks, energy drinks, juice/fruit drinks, iced teas/lemonades, coffee beverages, waters and milk/soya beverages. These included SSB subject to the NL tax (regular pop, energy drinks and fruit-flavoured drinks), and other beverages not subject to the tax (diet pop, juice, chocolate milk and regular milk). We collected prices for all purchase units, including single (e.g. 355 mL, 1 L) and bulk items (e.g. 12 cans of 355 mL). Regular prices were recorded, along with sale (discounted) prices if applicable. When tested for interrater reliability, the audit form demonstrated good reliability for identifying beverages available in store (kappa = 0·752 [0·711 to .792]), excellent reliability for recording regular prices (intraclass correlations [ICC] = 0·998 [0·997−0·998]) and sale prices (ICC = 0·999 [0·998 to 0·999] and moderate-to-good for identifying whether the beverage was on sale (kappa = 0·604 [0·477 to 0·731]). All prices were converted to price per 100 mL for comparability across products and time.

Research assistants also recorded product placement of any ready-to-drink beverages (not limited to the 53 beverages), such as placement at checkout, at ‘islands’, or on the ends of aisles in stores. Specifically, we counted the number of taxable and non-taxable beverage products by unique purchase unit placed at end of aisles, as islands in-store and at checkouts. Different flavours of the same product were counted only once. Further, any promotional signs for ready-to-drink beverages or beverage brands posted within the store or outside on the store property were recorded. For product placement and promotional signs, the research assistant recorded the product, brand, purchase unit and sale price (if applicable). Photos were taken to verify the data. Interrater reliability tests revealed excellent reliability in the number of promotions identified per store (ICC = 0·903 [95 % CI: 0·284, 0·990]). Due to the wide 95 % CI of the ICC, we removed the audits with the highest and lowest counts of promotions to reduce the risk of rater error. After these exclusions and retaining only stores with at least one audit in both pre-tax and post-tax periods, we included a total of 8056 promotions remained from 256 audits (131 pre-tax and 125 post-tax) from forty-one stores.

### Data analysis

#### Price change

For in-store beverage prices, we estimated the potential changes in posted regular prices and slope among taxable SSB compared with non-taxable beverages in NL, following the implementation of the NL SSB tax with an uncontrolled interrupted time series models fit with generalised least squares and auto-regressive moving average correlation structures. Estimates were adjusted for potential confounders including geography (rural and urban), store ownership and bulk purchase units. Prior to analysis, consumer price indices were used to calculate monthly inflation factors up August 2023 (the final month of data collection), which were used to adjust prices to be comparable to August 2023 in order to ensure any price changes detected would not be attributable to the changes expected to be caused by inflation.

#### Discount pricing

To examine changes in the proportion of taxable SSB and non-taxable beverages (comparison) that were discounted pre- and post-tax implementation, we performed Pearson *χ* ^2^ tests of independence first solely on the association between sale promotion status (sale *v*. no sale) and time period (pre- or post-tax implementation), then repeated with beverage type, store type and purchase unit (single unit *v*. bulk pack) as layer (stratification) variables to examine their effect on the relationship.

To examine whether the size of the discount (percent discounted from regular price) changed between pre-tax and post-tax time periods in taxable SSB and non-taxable beverages, multiple linear regressions were used to examine how discount size was affected by time (pre-tax *v*. post-tax) with beverage type, store type and purchase unit as covariates. These analyses were performed separately for taxable and non-taxable beverages.

#### Product placement and promotional signs

To assess changes in the frequency of ‘promotions’ (product placement and promotional signs) of taxable SSB and non-taxable beverages, linear mixed models were used. We used 2 × 2 tables and Pearson *χ* ^2^ tests of independence to assess potential changes in the proportion of promotions for both taxable and non-taxable beverages between pre-tax and post-tax periods, overall and by beverage category.

## Results

Data were collected for a total of 25 929 beverages (*n* 14 372 pre-tax; *n* 11 557 post-tax) (see Supplement B, Table B). Stores varied in their display of price tags where some had few or no prices, some displayed prices clearly for all beverages, and some displayed sale prices in ways that obscured regular prices. Regular prices were visible and recorded for 8,565 beverages pre-tax (60 %) and 10 317 post-tax (89 %), with most coming from convenience (43 %) and grocery stores (42 %), and stores located in urban areas (88 %) (Table 1). Taxable SSB comprised over half of the beverage sample (56 %). Sale prices were visible and recorded for 8022 beverages (*n* 4354 pre-tax; *n* 3668 post-tax).

**Table 1.** Sample characteristics of in-store retail beverage price data before and after NL SSB tax implementation

	Overall, *n* 18 882		Pre-tax, *n* 8565		Post-tax, *n* 10 317	
Characteristic	%	*n*	%	*n*	%	*n*
**Store type**						
Convenience	43	8109	43	4479	42	3630
Grocery	42	7959	42	4287	43	3672
Drug	11	2145	12	1196	11	949
Dollar	3·5	669	3·4	355	3·7	314
**Beverage grouping**						
SSB, taxable	56	10 605	56	5822	56	4783
Diet	30	5709	29	3037	31	2672
Unsweetened	12	2259	12	1280	11	979
SSB, non-taxable	1·6	309	1·7	178	1·5	131
**Purchase unit**						
Single unit	73	13 853	74	7616	73	6237
Bulk pack	27	5029	26	2701	27	2328
**Urbanicity**						
Urban	88	16 546	91	9430	83	7116
Rural	12	2336	8·6	887	17	1449

SSB, sugar-sweetened beverage.

### Price change pre- and post-tax implementation

The unadjusted time series analysis found no evidence of a price change in posted prices of taxable SSB in-store between the years before and after the tax was implemented in NL, compared to non-taxable beverages. No differences were found in the posted pre-tax price (*β* = −0·00019, 95 % CI [0·00092, 0·00054], *P* = 0·605), price when the tax was implemented (*β* = 0·0039, 95 % CI [0·022, 0·030], *P* = 0·77) or post-tax price (*β* = 0·00014, 95 % CI −0·00088 −0·0012, *P* = 0·783) (Table 2). This remained consistent when the model was adjusted considering store type, urbanicity or single *v*. multi-pack purchase units (Table 2).

**Table 2.** Unadjusted and adjusted differences in the in-store price per 100 mL of SSB, subject to the NL tax (taxable) before and after implementation of the NL SSB tax

	Unadjusted model			Adjusted model		
	*β*	95 % CI	*P* value	*β*	95 % CI	*P* value
Weeks pre-tax*taxable SSB (pre-intervention slope difference)	−0·00019	−0·00092, 0·00054	0·605	0·000036	−0·00068, 0·00075	0·921
Taxable SSB*post-tax period (immediate price difference)	0·0039	−0·022, 0·030	0·770	0·002	−0·023, 0·028	0·876
Taxable SSB*weeks post-tax (post-intervention slope difference)	0·00014	−0·00088, 0·0012	0·783	−0·000035	−0·0010, 0·00097	0·945
Intercept	0·53	0·48, 0·58	<0·001	0·46	0·42, 0·50	<0·001
Pre-intervention time (week)	−0·0016	−0·0032, −0·0000051	0·0493	−0·0009	−0·0018, 0·0000053	0·0514
Taxable SSB^a^	0·033	0·010, 0·056	0·0043	0·031	0·0086, 0·053	0·00652
Post-tax time period^b^	−0·047	−0·10, 0·0078	0·0926	−0·057	−0·089, −0·026	<0·001
Post-intervention time (week)	0·0028	0·00058, 0·0050	0·0134	0·0023	0·0010, 0·0036	<0·001
Convenience store^c^			0·2	0·18, 0·21	<0·001	
Dollar store^c^			−0·11	−0·15, −0·064	<0·001	
Drug store^c^			0·049	0·024, 0·075	<0·001	
Bulk pack purchase unit^d^			−0·12	−0·13, −0·11	<0·001	
Urban region^e^			−0·011	−0·035, 0·012	0·349	

SSB, sugar-sweetened beverage.

a Reference category: Non-taxable beverages.

b Reference category: Pre-tax time period.

c Reference category: Grocery store.

d Reference category: Single unit.

e Reference category: Urban region.

### Discount pricing

#### Proportion of beverages discounted

Across all time, 38·3 % of beverages (*n* 9934) were discounted; 39·1 % of taxable SSB were discounted compared with 37·2 % of non-taxable beverages. The proportion of taxable SSB discounted was significantly higher in the post-tax year (40·5 %) than in the pre-tax year (38·0 %) (*χ* ^2^ = 9·693, *P* = 0·002). There was no significant change in the proportion of non-taxable beverages discounted between the post-tax year (37·7 %) and pre-tax year (36·9 %) (*χ* ^2^ = 0·874, *P* = 0·350).

By beverage category, there was a significant increase in the proportion of taxable energy drinks (43·7 % *v*. 49·6 %, *χ* ^2^ = 7·079, *P* = 0·008) and taxable iced teas and lemonades (20·1 % *v*. 25·8 %, *χ* ^2^ = 0·5273, *P* = 0·022) that were discounted in the post-tax year compared with pre-tax. There were no significant differences in the proportion of non-taxable beverages on discount, except for non-taxable waters, which significantly decreased from 32·9 % to 27·9 % (*χ* = 5·686, *P* = 0·018). There were no other significant changes in the proportion of other beverage categories that were discounted (Table 3).

**Table 3.** Comparison of beverage types on sale pre-tax *v*. Post-Tax with *χ* ^2^ test statistic, taxable and non-taxable beverages

Beverage types (*n*)	Sale status	Taxable (*n* 14 632)				Non-taxable (*n* 11 297)			
		Pre-tax	Post-tax	*χ* ^2^	*P* value	Pre-tax	Post-tax	*χ* ^2^	*P* value
Pop *n* 14 171	**Sale**	41·3 %	42·4 %	0·998	0·320	42·0 %	43·8 %	1·591	0·208
	**No Sale**	58·7 %	57·6 %			58·0 %	56·2 %		
Sports drink *n* 2,456	**Sale**	35·5 %	36·6 %	0·229	0·638	38·6 %	37·6 %	0·082	0·776
	**No Sale**	64·5 %	63·4 %			61·4 %	62·4 %		
Energy drink *n* 3,524	**Sale**	**43·7 %**	**49·6 %**	**7·079**	**0·008** *	46·0 %	48·8 %	1·157	0·282
	**No Sale**	**56·3 %**	**50·4 %**			54·0 %	51·2 %		
Juice/Fruit drink *n* 868	**Sale**	15·9 %	23·2 %	2·724	0·115	13·4 %	14·4 %	0·111	0·801
	**No Sale**	84·1 %	76·8 %			86·6 %	85·6 %		
Iced tea/Lemonade *n* 1,386	**Sale**	**20·1 %**	**25·8 %**	**5·273**	**0·022** *	24·0 %	30·8 %	1·364	0·300
	**No Sale**	**79·9 %**	**74·2 %**			76·0 %	69·2 %		
Coffee drink^a^ *n* 305	**Sale**	16·5 %	18·9 %	0·289	0·638	–	–	–	–
	**No Sale**	83·5 %	81·1 %			–	–		
Water *n* 2,255	**Sale**	37·0 %	34·8 %	0·162	0·721	**32·9 %**	**27·9 %**	**5·686**	**0·018** *
	**No Sale**	63·0 %	65·2 %			**67·1 %**	**72·1 %**		
Milk and soya milk^a^ *n* 964	**Sale**	–	–	–	–	19·9 %	19·7 %	0·005	1·000
	**No Sale**	–	–			80·1 %	80·3 %		
Total *n* 25 929	**Sale**	**38·0 %**	**40·5 %**	**9·693**	**.002** *	36·9 %	37·7 %	0·874	0·358
	**No Sale**	**62·0 %**	**59·5 %**			63·1 %	62·3 %		

* Denotes statistically significant *P* value. Bolded values indicate a significant change in proportion of taxable beverages on sale was observed.

a No data collected in sample for non-taxable coffee drinks or taxable milk/soya milks.

Significant results from multiple *χ* ^2^ tests stratified by beverage type, store type, and purchase unit for the pre-tax *v*. post-tax years are summarised in Supplement B, Table C.

#### Size of the discount

The size of the discount (depth of sale) of the sale price compared with the regular price was larger (i.e. more discounted) at post-tax for both taxable SSB (pre-tax: 22·64 % [12·49] *v*. post-tax: 26·00 % [15·90]) and non-taxable beverages (pre-tax: 22·52 % [12·01] *v*. post-tax: 26·36 % [16·38]).

There was a significant effect of time period (pre- *v*. post-tax years) on percent discount with taxable SSB (*F* = 32·014, sig. <0·001; *R*-squared = 0·071, adj. *R*-squared = 0·069). Taxable SSB were discounted 3·2 % more at post-tax than at pre-tax (*B* = −0·032 [0·004], sig. < 0·001) (Table 4). A similar significant effect was seen with non-taxable beverages (*F* = 23·811, sig. < 0·001; *R*-squared = 0·072, adj. *R*-squared = 0·069). Non-taxable beverages were discounted 3·9 % more at post-tax than at pre-tax (*B* = −0·039 [0·005], sig. < 0·001) (Table 4).

**Table 4.** Multiple linear regression for effect of time period (pre- *v*. Post-Tax implementation) on percent discount, controlling for purchase unit, beverage type and store type

Comparing pre-tax year *v*. post-tax year										
	Taxable beverages					Non-taxable beverages				
	Unstandardised Coefficients		Standardised Coefficients	*t*	Sig.	Unstandardised Coefficients		Standardised Coefficients	*t*	Sig.
	*B*	Std. Error	Beta			*B*	Std. Error	Beta		
Constant	−0·199	.005		−42·217	<.001*	−.198	.006		−35·189	<.001*
**Post-tax** ^a^	**−.032**	**.004**	**−.113**	**−7·987**	**<.001** ^*^	**−.039**	**.005**	**−.136**	**−8·188**	**<.001** ^*^
Bulk pack^b^	.020	.005	.063	3·832	**<.001** ^*^	.021	.006	.066	3·487	**<.001** ^*^
Sports drink^c^	−.030	.007	−.064	−4·376	**<.001** ^*^	−.052	.009	−.099	−5·766	**<.001** ^*^
Energy drink^c^	−.046	.006	−.122	−8·196	**<.001** ^*^	−.040	.007	−.110	−6·204	**<.001** ^*^
Juice/Fruit drink^c^	−.039	.019	−.030	−2·110	**.035** ^*^	−.045	.017	−.045	−2·651	**.008** ^*^
Tea/Lemonade^c^	.002	.010	.004	0·248	.804	.008	.019	.007	.416	.677
Coffee drink^c^	.020	.020	.015	1·008	.313	−.019	.007	−.045	−2·589	**.010** ^*^
Water^c^	−.024	.014	−.024	−1·704	.089	.025	.012	.036	2·092	**.037** ^*^
Convenience^d^	−.037	.005	−.129	−7·592	**<.001** ^*^	−.031	.006	−.110	−5·667	**<.001** ^*^
Drugstore^d^	−.065	.010	−.099	−6·742	<.001*	−.063	.011	−.096	−5·581	**<.001** ^*^
Dollar store^d^	−.120	.049	−.035	−2·455	.014*	−.038	.044	−.015	−.874	.382

* Denotes statistically significant *P* value. The post-tax time period variable and values have been bolded to emphasize the significant effect observed of post-tax time period on percent discount.

a Reference category = Pre-tax year.

b Reference category = Single unit.

c Reference category = Pop/Soda.

d Reference category = Grocery store.

e Reference category = Immediately pre-tax implementation (August 2022).

### Placement and promotional signage

A total of 8056 in-store promotions were recorded, 3979 (49·4 %) at pre-tax and 4077 (50·6 %) at post-tax. Price discounts were included for more than half (53·0 %) of recorded product placements and promotional signs. More than half of the promotions (61·9 %) were product placements; the remaining were promotional signs. Product placement of beverages was found at island displays (42·1 %, *n* 2102), at checkout (31·0 %, *n* 1548), and at the end of aisles (26·8 %, *n* 1338) in stores. Most promotional signs were found inside the store (91·2 %, *n* 2797). More than half of promotions were found in convenience stores (57·9 %) (*n* 4664), with 39·1 % (*n* 3149) from grocery stores and 3·0 % (*n* 243) from drug stores.

Taxable SSB were more promoted than non-taxable beverages at both time points, with the mean number increasing more for taxable SSB than non-taxable beverages in the post-tax year. Taxable SSB comprised 60·6 % (*n* 2413/3979) of the promotions pre-tax and 61·2 % (2492/4073) post-tax. There was no change in the contribution of taxable *v*. non-taxable beverages to promotions before and after the tax (*χ* ^2^ = 0·247, *P* = 0·631) overall.

Soft drinks were the most promoted before and after the tax was implemented, where regular soft drinks made up 58–61 % of all promotions and diet soft drinks made up 43–29 % of all promotions. Sugar-sweetened energy drinks (22–25 %) and sugar-sweetened sports drinks (7–8 %) were the other most common taxable SSB promoted. Waters (25–31 %), followed by sugar-free energy drinks (14–15 %), and sugar-free sports drinks (3–6 %) were the most common non-taxable beverages promoted. There was no difference in the frequency of promotions of any type of beverage, except for sports drinks (*χ* ^2^ = 8·203, *P* = 0·004) and fruit juices and drinks (*χ* ^2^ = 43·818, *P*<0·001) (Table 5).

**Table 5.** Frequency of product placement and promotional signs for taxable and non-taxable beverages by beverage type, pre- and post-tax implementation

	Taxable beverages				Non-taxable beverages				***χ*** ^**2**^	
	Pre-tax		Post-tax		Pre-tax		Post-tax			
Beverage type	*n*	%	*n*	%	*n*	%	*n*	%		***P*** **value**
Soft drinks	1397	57·9%	1531	61·4%	670	42·8%	778	49·2%	0·807	0·385
Sports drinks	**179**	**7·4%**	**200**	**8·0%**	**48**	**3·1%**	**96**	**6·1%**	**8·203**	**0·004** ^*^
Energy drinks	594	24·6%	553	22·2%	216	13·8%	229	14·5%	1·353	0·264
Fruit juices and drinks	**17**	**0·7%**	**64**	**2·7%**	**45**	**2·9%**	**13**	**0·8%**	**43·818**	**<0·001** ^*^
Lemonades and iced teas	94	3·9%	67	2·7%	9	0·6%	6	0·4%	0·015	1·000
Coffee drinks^a^	26	1·1%	10	0·4%	–		–		–	
Waters	50	2·1%	50	2·0%	487	31·1%	391	24·7%	1·084	0·340
Milk and soya milks^a^	–		–		42	2·7%	60	3·8%	–	
Other	56	2·3%	17	0·7%	49	3·1%	8	0·5%	1·764	0·262
Total	2413	100·0%	2492	100·0%	1566	100·0%	1581	100·0%	0·247	0·631

* Denotes statistically significant *P* value. Bolded values indicate a significant change in proportion of taxable beverages on sale was observed.

a No data collected in sample for non-taxable coffee drinks or taxable milk/soya milks.

**Figure 1.**
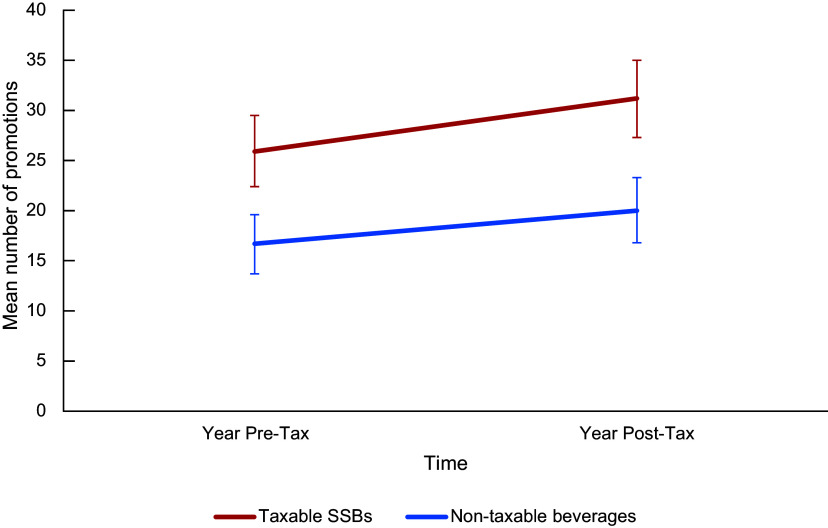
Mean number of in-store promotions for taxable SSB and non-taxable beverages, years pre- and post-tax implementation. Error bars represent 95 % CI. SSB, sugar-sweetened beverages.

The mean (95 % CI) number of promotions of taxable SSB increased from the year before to the year after the tax (mean difference: +5·2 [−0·1, 10·5], *F* = 3·789, *P* = 0·053); however, this change was only approaching statistical significance. The change in number of promotions was lesser and non-significant for non-taxable beverages (mean difference 3·4 [–1·0, 7·7], *F* = 2·268, *P* = 0·134) (Figure 1).

## Discussion

This study evaluated real-life impacts of the NL SSB tax 1 year after its implementation, examining changes in beverage pricing and marketing. Results show a potential increase in some beverage marketing efforts following the implementation of a $0·20 per liter excise tax on SSB in NL, though no change in in-store shelf prices of taxable SSB compared with non-taxable beverages was observed. The proportion of discounted taxable SSB was significantly higher in the year following tax implementation compared with the year prior, but no significant change was found for non-taxable beverages. The size of the discount for beverages on sale was similar for both taxable and non-taxable beverages. No significant difference was found for frequency of promotions of taxable SSB, though an insignificant increase was observed both immediately after tax implementation and the year following – this may possibly be explained by the small sample of stores included and high variability of promotion outcomes. Promotions of taxable SSB were more frequent than non-taxable beverages, both before and after tax implementation.

Zenk et al.^(26)^ evaluated changes in price promotions, depth of sale and advertising following SSB tax implementation in Oakland, California, comparing with Sacramento. Despite reporting short-term reductions at 6- and 12-months post-tax in prevalence of price promotions for SSB overall and regular soda, by 2 years post-tax these differences were no longer observed. No significant changes 2 years post-tax were observed in odds of price promotions (ROR = 0·97 [0·65, 1·44]), advertising (interior ROR = 1·33 [0·66,2·68]; exterior ROR = 0·84 [0·48,1·47]), or depth of sale (coefficient = 0·13 [−0·13, 0·39]) for SSB, nor for untaxed beverages. This suggests that, despite potential short-term changes, long-term effects on marketing practices may not be sustained in response to SSB taxes.^(26)^ Zenk et al.^(26)^ theorised that initial reductions in price promotions served to instead offset revenue loss. Our results show a different initial response to the tax in NL, with prevalence of SSB price promotions increasing following tax implementation. However, our study includes only data up to 1-year post-tax, so it is unknown whether this will lead to sustained shifts in retail marketing practices. Further, changes in promotions in our study were not uniform across beverage types with significant changes seen in some beverage categories (e.g. energy drinks and iced teas/lemonades) and not other beverage categories (e.g. soft drinks). While explanations for the lack of change in soft drink promotions remain speculative, the higher-than-expected revenue generated by the tax,^(27)^ as well as cultural norms in the province, imply that soft drinks continued to sell well despite the introduction of the tax or effects of seasonality – limiting the need for increased promotions. With soft drinks as a major product in the beverage market, a lack of marketing changes amongst this notable beverage category may suggest that any marketing changes seen in this study were attributable to factors other than attempts by the food industry to combat the NL SSB tax.

Another study by El-Sayed & Powell^(28)^ analysed price promotions in Oakland along with the comparison region of Sacramento, considering 2 years post-tax, and found no significant changes in prevalence of price promotions for SSB relative to the comparison region, though depth of discount increased significantly. It is hypothesised that the increase in amount by which SSB was discounted following implementation of the Oakland SSB tax may reflect a strategy used by manufacturers to counteract the impact of the tax.^(28)^ We did find an increase in prevalence of price promotion for taxable beverages, though depth of discount increased for both taxable and non-taxable beverages post-tax. Without a comparison region, it is not possible to determine conclusively whether these changes are different than normal variations in beverage markets. Further studies in Philadelphia^(29)^ and Seattle^(30)^ found an increase in in-store SSB advertising, and mixed evidence for changes in marketing displays in response to SSB taxes, respectively.

A study by Keller et al.^(31)^ evaluated changes to marketing strategies, including promotional frequency, discount depth and feature promotions in store circulars (flyers), as well as the effectiveness of each of these tactics in response to SSB taxes implemented in five USA markets (Philadelphia, Boulder, Oakland, San Francisco and Seattle) over 1 year. As observed in prior research, sales volume decreased while prices increased post-tax introduction.^(31)^ Data from over 1500 stores showed that SSB taxes impact the effectiveness of marketing tactics on SSB sales. Flyer features and discounts were less effective on consumer behaviour with SSB taxes in place (–17 % and –30 %, respectively). Keller et al.^(31)^ argued that in the context of an SSB tax, the usual depth of sale will be less effective at improving sales and deeper discounts are needed to achieve the same consumer effect. In contrast, the frequency of promotions tended to significantly increase its effectiveness on consumer behaviour in the presence of an SSB tax (+13·7 %) explained by “the notion that consumers shift their purchases from regular prices toward promotions to counteract steep (regular) price increases due to the soda tax” (p.404).^(31)^ Despite this, findings show that retailers adapted marketing strategies by decreasing promotional frequency, promotional depth and feature promotions – elements that serve to exacerbate the negative sales effect of SSB taxes on consumers.^(31)^ This illustrates that SSB tax-related changes in effectiveness of marketing strategies on consumers and actual SSB marketing conduct adaptations are not always in alignment, perhaps contributing to the explanation of inconsistent marketing responses post-tax introduction.^(31)^ While evidence from Keller et al.^(31)^ shows that SSB taxes can be effective in reducing SSB sales, they emphasises the need for consideration of not only the direct effects of these taxes, but also indirect effects of industry response as marketing and promotional conduct play an undeniable role in consumer purchasing behaviour and policy outcomes.

Forde et al.^(18)^ hypothesised a theoretical framework that describes how companies make marketing changes in response to sugar taxes and how spillover effects involve changes in products, prices, promotion or placement − the ‘four Ps’ or ‘marketing mix,’ following the introduction of the UK Soft Drinks Industry Levy (SDIL). Notably, the design of the SDIL, a tiered tax on soft drink manufacturers and importers according to sugar content, differs from the NL tax.^(18)^ As stated by these authors, neither they – nor our research group – are aware of other published studies on changes across the four Ps in response to implementation of a tax on sugary beverages. Our study focuses on all Ps using quantitative data, while the Forde et al. study is qualitative in nature. Forde et al.^(18)^ observed that heterogeneity in the impact of the SDIL on beverage marketing was explained in part by individual company context. Their framework includes sugary beverage taxes like the SDIL as one of many factors considered that determine industry marketing changes – which may result in no change, or changes either conflicting or aligning with public health goals. It is stated that in this case, decision-making surrounding marketing changes was accelerated rather than precipitated by the tax, given the constant cycle of evaluation illustrated by the theoretical framework. Evidence suggests that certain industry impacts of the SDIL are small or short-lived, consistent with other emerging literature on this^(18)^ and other SSB taxes.^(26)^ Authors emphasise the importance of exploring potential commercial responses in conjunction with public health goals, which can inform design of taxation policies more robust to industry resistance and counteraction to maximise effectiveness.^(18)^

Regions including Mexico and Chile have regulations surrounding advertising of SSB in addition to taxes. In 2014, Chile implemented a tax reform increasing rates for high-sugar SSB,^(32)^ and in 2016 banned child-targeted television advertising of SSB; a daytime advertising ban followed in 2019.^(33)^ From pre-regulation to the 2019 ban, total ads for targeted products decreased by 61·8 percentage points, showing the effectiveness of mandatory marketing restrictions.^(33)^ In 2013, Mexico published a framework for policies to combat obesity, followed by implementation of an SSB tax and regulations banning SSB advertising during children’s programming, allowing only advertising of nutritious foods/beverages.^(34)^ Several studies have provided evidence of the resulting high tax pass-through to consumers, reductions in purchases and sales of SSB and increases in water sales.^(34)^ Estimates of the long-term impact show benefits in reducing BMI and other risk factors, with the potential of reductions in diabetes, stroke and myocardial infarction – thereby decreasing both morbidity and mortality rates and healthcare costs.^(34)^ While evidence-based and seemingly effective, there appear to be few cases where both policies are enacted in unison. Although different in context, the examples from Mexico and Chile provide an example of health promoting policy suites that can be considered for broader, stronger impacts on healthy eating.

Existing literature implies that the potential changes in marketing observed in response to SSB taxes may counteract effects on SSB purchasing and consumption, thus diminishing their effectiveness with regard to behaviour change and public health goals. The lack of change in posted prices of taxable SSB observed in this study indicates that the NL SSB tax was not clearly communicated to consumers at the point of decision-making. Our results do not represent the actual economic pass-through of the tax but rather the visibility of the tax at point of decision-making for consumers; that is, while our unpublished research suggests that most stores were charging the (partial or whole) cost of the tax to consumers by applying the tax at checkout, we did not detect a change in the visible posted prices – which is what customers see when deciding to add items to their carts. The same phenomenon was seen in our study of online SSB prices in NL, where no change in visible ‘shelf’ prices (pre-checkout) was found post-tax.^(35)^ It is notable that the NL policy lacks requirements about how – and where – the tax is to be communicated to consumers, indicating that any communication of the tax is decided by retailers and is not required to be posted on the ‘shelf’ tag.^(13)^ Previous research suggests that displaying a retail tax on the shelf may be more influential to consumers than when applied at point-of-sale, as purchasing decisions are generally based on the shelf tags where price increases are not communicated – unless this is mandated in and enforced by the policy.^(36)^ When not visible at point of decision-making, the consumer may have much lower awareness or consideration of the tax, which may negatively influence its effectiveness as a health-promoting intervention by negating the purpose of the tax and its intended behavioural effects.

While some marketing changes post-tax were observed in this study, results should be interpreted cautiously as they cannot be attributed solely or definitively to the NL SSB tax. A lack of marketing changes by industry observed in NL after the SSB tax was introduced, coupled with lack of price change shown on shelves, may suggest that many retailers opted not to communicate the cost of the tax to consumers at point of decision-making rather than actively attempting to counteract it. This may also potentially reflect a lack of perceived threat of the NL SSB tax to industry, considering its small contribution to regional and national markets. Marketing tactics have been shown to be impactful on interventions such as taxation, and while excise taxes may produce short-term changes, they alone may not be sufficient to drive lasting changes in the marketing, sales and therefore consumption of unhealthy food and beverages. Future studies should aim to evaluate if any marketing changes (or lack thereof) observed here are sustained beyond the first year post-tax and how these marketing strategies relate to potential changes in SSB consumption in NL, relative to a no-tax comparison region. These results also provide important insights and considerations for policymakers in the design or re-evaluation of SSB taxes.

### Strengths and limitations

This research is part of a larger study – the first to evaluate the impact of Canada’s first provincial SSB tax in NL. It contributes to highly limited evidence exploring changes in beverage marketing immediately following implementation of an SSB tax. Our research provides crucial context on industry marketing response, which is needed to fully understand the impact of the policy and to explain its potential effectiveness or ineffectiveness. Our results will help inform more in-depth future investigations into this and other SSB taxes, along with having important implications for policymakers. This research allows for real-life evaluation of an SSB tax and its effects, possessing high ecological validity.

The observational nature of the study causes challenges, namely lack of control over the intervention and extraneous factors, which impacts our ability to separate the effect of the tax from other factors that may impact beverage pricing and promotions. We also lack simultaneous pricing and promotions data from a comparable control location, thus rendering us unable to account for secular changes across the study’s time period. These limitations should be carefully considered when interpreting our results. Additionally, we noted high variability in promotion outcomes across a relatively small store sample, which may have affected our ability to detect statistical significance. It is also of note that visibility of beverage prices and the SSB tax was less than expected contributing to missing data, representing a lack of clear communication to consumers of the tax despite highly visible displays of beverage promotions. While inclusion of pre-checkout price data indicates the degree of communication of the tax, it does not fully represent the actual price paid by the consumer, or the economic pass-through of the tax. Finally, we recognise that seasonal differences may have an impact on beverage marketing, and the differences in the months included in data collection periods for pre- and post-tax has the potential to introduce bias.

## Conclusion

Following the introduction of Canada’s first provincial SSB tax in NL in 2022, our findings show limited changes in beverage marketing post-tax. Notably, they indicate no change in posted beverage prices, signalling poor communication of the tax to consumers and thus the potential to render the policy ineffective as a public health intervention. A regulatory gap in this policy may exist that enables retailers to display the tax in-store at their own volition. We noted a significant increase in the proportion of taxable SSB that were discounted in the post-tax year compared with pre-tax. The increase in the frequency of product placement and promotional signs of taxable SSB was insignificant. Although we cannot definitively link any changes to the NL SSB tax due to the nature of the study design, our findings provide useful insight for future investigation. Industry response to SSB taxes is an important factor in the effectiveness of these policies in promoting healthy behaviour change in the population. Potential increases in marketing efforts of SSB following tax introduction can, in some cases, serve to counteract public health goals. When designing or evaluating intentions aimed at reducing SSB intake, policymakers should aim to understand and consider the crucial role that beverage marketing plays in the success of interventions achieving intended effects.

## Supporting information

Dooley et al. supplementary material 1 Dooley et al. supplementary material

Dooley et al. supplementary material 2 Dooley et al. supplementary material

## Supplementary material

To view supplementary material for this article, please visit https://doi.org/10.1017/S1368980026102146.
