# Supplementary material for: In-store beverage pricing and marketing before and after a sugar-sweetened beverage tax in Newfoundland and Labrador
Source: Public Health Nutr. 2026 Feb 16;29(1):e58. doi: 10.1017/S1368980026102146 (PMC13112304; doi:10.1017/S1368980026102146)
Supplement: Dooley et al. supplementary material 1 — Dooley et al. supplementary material [file S1368980026102146sup001.docx]

**SUPPLEMENT B**

Table B: Numbers of beverages from pre- and post-tax data collection periods, taxable and non-taxable, by beverage type.

|  | **Taxable Beverages** | | | **Non-Taxable Beverages** | | |
| --- | --- | --- | --- | --- | --- | --- |
|  | **Pre-tax** | **Post-tax** | **Total** | **Pre-tax** | **Post-tax** | **Total** |
| **Beverage Type** | | | | | | |
| Pop | 4925 | 3984 | **8909** | 2874 | 2388 | **5262** |
| Sports drink | 910 | 704 | **1614** | 459 | 383 | **842** |
| Energy drink | 1117 | 909 | **2026** | 809 | 689 | **1498** |
| Juice/Fruit drink | 176 | 142 | **318** | 328 | 222 | **550** |
| Tea/Lemonade | 673 | 481 | **1154** | 125 | 107 | **232** |
| Coffee drink | 194 | 111 | **305** | 0 | 0 | **0** |
| Water | 165 | 141 | **306** | 1059 | 890 | **1949** |
| Milk | 0 | 0 | **0** | 558 | 406 | **964** |
| **Total** | **8160** | **6472** | **14632** | **6212** | **5085** | **11297** |

**Discount Pricing – Proportion on sale**

Table C: Summary table for statistically significant factors for proportion of beverages on sale using multiple stratified Chi-Square tests, year pre-tax vs post-tax and rounds immediately pre-tax vs post-tax, taxable and non-taxable beverages.

| Time period | Significant factors | χ^2^ | p-value | Proportion on sale, pre-tax | Proportion on sale, post-tax | Percent change |
| --- | --- | --- | --- | --- | --- | --- |
| **Taxable Beverages** (n=14,632) | | | | | | |
| Pre-tax vs Post-tax years | Energy drink  Iced tea/Lemonade  Convenience store  Single unit | 7.079  5.273  9.690  10.911 | .008  .022  .002  <.001 | 43.7%  20.1%  40.4%  37.2% | 49.6%  25.8%  44.0%  40.3% | + 5.9%  + 5.7%  + 3.6%  + 3.1% |
| Rounds 5 (pre-tax) vs 6 (post-tax) | Pop  Convenience  Drugstore  Single unit | 6.967  10.860  3.906  9.416 | .008  <.001  .048  .002 | 41.9%  44.4%  8.0%  39.6% | 37.2%  38.0%  15.5%  34.6% | - 4.7%  - 6.4%  + 7.5%  - 5.0% |
| **Non-Taxable Beverages** (n=11,297) | | | | | | |
| Pre-tax vs Post-tax years | Water | 5.686 | .017 | 32.9% | 27.9% | - 5.0% |
| Rounds 5 (pre-tax) vs 6 (post-tax) | Sports drink  Convenience store  Drugstore  Single unit | 5.394  10.668  3.961  6.187 | .020  .001  .047  .013 | 41.4%  45.7%  6.9%  38.2% | 27.7%  38.5%  15.2%  33.7% | - 13.7%  - 7.2%  + 8.3%  - 4.5% |

**Discount Pricing – Percent Discounted**

Table D: Mean percent discount for beverages on sale pre- and post-tax, by beverage type, store type, and purchase unit.

|  | **Taxable Beverages**  Mean (SD) | | **Non-Taxable Beverages**  Mean (SD) | |
| --- | --- | --- | --- | --- |
|  | **Pre-tax** | **Post-tax** | **Pre-tax** | **Post-tax** |
| **Beverage Type** (n) | | | | |
| **Pop**  n=4669 | -.2163 (.1317) | -.2418 (.1561) | -.2156 (.1271) | -.2393 (.1611) |
| **Sports drink**  n=765 | -.2345 (.1176) | -.2933 (.1706) | -.2392 (.1133) | -.3367 (.1861) |
| **Energy drink**  n=1444 | -.2655 (.1098) | -.3070 (.1562) | -.2545 (.1073) | -.3004 (.1540) |
| **Juice/Fruit drink**  n=125 | -.2657 (.1286) | -.2285 (.1089) | -.2630 (.1098) | -.2852 (.1243) |
| **Tea/Lemonade**  n=282 | -.2051 (.0870) | -.2430 (.1471) | -.1781 (.1105) | -.2523 (.2010) |
| **Coffee drink ^a^**  n=50 | -.1724 (.0830) | -.2361 (.1080) | - | - |
| **Water**  n=557 | -.2379 (.0767) | -.2911 (.1733) | -.2253 (.1164) | -.2712 (.1558) |
| **Milk ^a^**  n=152 | - | - | -.1865 (.0833) | -.2327 (.1451) |
| **Store Type** (n) | | | | |
| **Grocery**  n=3643 | -.1987 (.1230) | -.2287 (.1687) | -.2020 (.1187) | -.2336 (.1758) |
| **Convenience**  n=3978 | -.2452 (.1160) | -.2880 (.1469) | -.2397 (.1128) | -.2893 (.1498) |
| **Drugstore**  n=402 | -.2907 (.1566) | -.2652 (.1321) | -.2804 (.1495) | -.2771 (.1442) |
| **Dollar store**  n=21 | -.5000 (0)^b^ | -.2123 (.1351) | -.5000 (0)^c^ | -.2294 (.1464) |
| **Purchase Unit** (n) | | | | |
| **Single unit**  n=5838 | -.2420 (.1238) | -.2677 (.1409) | -.2402 (.1186) | -.2697 (.1437) |
| **Bulk pack**  n=2206 | -.1857 (.1187) | -.2404 (.1965) | -.1833 (.1142) | -.2476 (.2068) |

^a^ No data collected in sample for non-taxable coffee drinks or taxable milk/soy milks.

^b^ Based on only 4 observations.

^c^ Based on only 2 observations.

**Product Placement and Promotional Signs**

Table E: Mean product placement and promotional signs for taxable and non-taxable beverages, years before and after the implementation of the NL SSB tax.

|  | **Taxable beverages** | | | | **Non-taxable beverages** | | | |
| --- | --- | --- | --- | --- | --- | --- | --- | --- |
|  | **Pre-tax** | **Post-tax** |  | | **Pre-tax** | **Post-tax** |  | |
|  | **Mean**  **[95% CI]** | **Mean [95% CI]** | **Mean Diff.**  **[95% CI]** | **F (p-value)** | **Mean**  **[95% CI]** | **Mean**  **[95% CI]** | **Mean Diff.**  **[95% CI]** | **F (p-value)** |
| Total Product Placement and Promotional Signage | 25.9  [22.4, 29.5] | 31.2  [27.3, 35.0] | +5.2  [-0.1, 10.5] | 3.789 (0.053) | 16.7  [13.7, 19.6] | 20.0  [16.8, 23.3] | +3.4  [-1.0, 7.7] | 2.268 (0.134) |
