# Supplementary material for: In-store beverage pricing and marketing before and after a sugar-sweetened beverage tax in Newfoundland and Labrador
Source: Public Health Nutr. 2026 Feb 16;29(1):e58. doi: 10.1017/S1368980026102146 (PMC13112304; doi:10.1017/S1368980026102146)
Supplement: Dooley et al. supplementary material 2 — Dooley et al. supplementary material [file S1368980026102146sup002.docx]

| Urban (St. John's Census Metropolitan Area) | |  |  |  |  |  |
| --- | --- | --- | --- | --- | --- | --- |
| **Store Type** | **Total Sampling Frame N** | **% of Store Type** | **% of Total** | **Sample n** | **% of Store Type** | **% of Sample** |
| **Grocery** | **35** |  | **17%** | **20** |  | **19%** |
| *National Chain* | 20 | 57% | 10% | 12 | 60% | 11% |
| *Independent* | 7 | 20% | 3% | 4 | 20% | 4% |
| *Provincial Chain* | 8 | 23% | 4% | 4 | 20% | 4% |
| **Convenience Store** | **128** |  | **63%** | **62** |  | **57%** |
| *National Chain* | 44 | 34% | 22% | 23 | 37% | 21% |
| *Independent* | 48 | 38% | 24% | 20 | 32% | 19% |
| *Provincial Chain* | 36 | 28% | 18% | 19 | 31% | 18% |
| **Drug Store** | **26** |  | **13%** | **13** |  | **12%** |
| *National Chain* | 16 | 62% | 8% | 8 | 62% | 7% |
| *Independent* | 10 | 38% | 5% | 5 | 38% | 5% |
| *Provincial Chain* | 0 | 0% | 0% | 0 | 0% | 0% |
| **Dollar Store** | **13** |  | **6%** | **13** |  | **12%** |
| *National Chain* | 13 | 100% | 6% | 13 | 100% | 12% |
| *Independent* | 0 | 0% | 0% | 0 | 0% | 0% |
| *Provincial Chain* | 0 | 0% | 0% | 0 | 0% | 0% |
| Total | 202 |  | 100% | 108 |  | 100% |

**SUPPLEMENT A**

Table A: Breakdown of proportionate stratified random sampling process for urban store sample selection.

**Urban Stores**  **Rural Stores**

Random stratified sample of rural communities, and random sampling of selected communities’ grocery and convenience stores

n = 26

Promotion audits completed

n = 41

Removed after high/low exclusions made ineligible^c^

*Excluded*

(n = 20)

Pricing audits completed

n = 80

No promotion audits completed^b^

*Excluded*

(n = 19)

No pricing or promotion audits completed^a^

*Excluded*

(n = 54)

Total stores

n = 134

Proportionate stratified sampling of grocery, convenience, drug, dollar stores

n = 108

Figure A: Summary flow chart for selection of stores and auditing process.

^a^ Stores with insufficient price data had zero audits completed, due to reasons such as store closures, store access denial, or inadequate staff capacity.

^b^ Stores with insufficient promotion data had fewer than one audit per period (pre-tax or post-tax).

^c^ For each store, audits with the highest and lowest counts of promotions were removed from the dataset to reduce risk of rater error. After removing the highest and lowest audits, 20 stores were excluded from analysis as they no longer had at least one audit at both pre-tax and post-tax times.
